# Modelling the correlation between EGFr expression and tumour cell radiosensitivity, and combined treatments of radiation and monoclonal antibody EGFr inhibitors

**DOI:** 10.1186/1742-4682-9-23

**Published:** 2012-06-19

**Authors:** Piernicola Pedicini, Rocchina Caivano, Barbara Alicia Jereczek-Fossa, Lidia Strigari, Barbara Vischioni, Daniela Alterio, Marta Cremonesi, Francesca Botta, Antonio Nappi, Giuseppina Improta, Giovanni Storto, Marcello Benassi, Roberto Orecchia

**Affiliations:** 1 I.R.C.C.S. C.R.O.B Regional Cancer Hospital, Rionero in Vulture, Italy; 2 U.O. of Radiotherapy, I.E.O. European Institute of Oncology, Milan, Italy; 3 University of Milan, Milan, Italy; 4 Laboratory of Medical Physics and Expert Systems, Regina Elena National Cancer Institute, Rome, Italy; 5 U.O. of Radiobiology, C.N.A.O, Pavia, Italy; 6 Service of Medical Physics, I.E.O. European Institute of Oncology, Milan, Italy; 7 Service of Medical Physics, Scientific Institute of Tumours of Romagna I.R.S.T, Meldola, Italy; 8 Department of Radiation Oncology, IRCCS CROB, 1 Padre Pio Street, 85028, Rionero in Vulture, PZ, Italy

## Abstract

**Purpose:**

To estimate the effects of heterogeneity on tumour cell sensitivity to radiotherapy combined with radiosensitizing agents attributable to differences in expression levels of Epidermal Growth Factor Receptor (*EGFr*).

**Materials and methods:**

Differences in radiosensitivity are not limited to cells of different cancer histotypes but also occur within the same cancer, or appear during radiotherapy if radiosensitizing drugs are combined with ionizing radiation. A modified biologically effective dose (*MBED*), has been introduced to account for changes in radiosensitivity parameters (*α* and *α/β*) rather than changes in dose/fraction or total dose as normally done with standard biologically effective dose *(BED)*. The *MBED* approach was applied to cases of EGFr over-expression and cases where EGFr inhibitors were combined with radiation. Representative examples in clinical practice were considered.

**Results:**

Assuming membrane *EGFr* over-expression corresponds to reduced radiosensitivity (*α*_*H*_ *= 0.15 Gy*^*-1*^ and *α*_*H*_*/β*_*H*_ *= 7.5 Gy*) relative to normal radiosensitivity (*α = 0.2 Gy*^*-1*^ and *α/β = 10 Gy*), an increased dose per fraction of 2.42 Gy was obtained through the application of *MBED*, which is equivalent to the effect of a reference schedule with *30* fractions of *2 Gy*. An equivalent hypo-fractionated regime with a dose per fraction of *2.80 Gy* is obtained if *25* fractions are set. Dose fractionations modulated according to drug pharmacokinetics are estimated for combined treatments with biological drugs. Soft and strong modulated equivalent hypo-fractionations result from subtraction of *5* or *10* fractions, respectively.

**Conclusions:**

During this computational study, a new radiobiological tool has been introduced. The *MBED* allows the required dose per fraction to be estimated when tumour radiosensitivity is reduced because *EGFr* is over-expressed. If radiotherapy treatment is combined with *EGFr* inhibitors, *MBED* suggests new treatment strategies, with schedules modulated according to drug pharmacokinetics.

## Background

Recently, radiobiology has been transformed thanks to new knowledge concerning cellular activation processes in response to an external stimulus. This knowledge has led to the identification of promising new drug therapies called "targeted therapy” [1].

Epidermal growth factor receptor (*EGFr*) has emerged as a central molecular target for modulation during cancer therapy. The correlation between over-expression of *EGFr* and clinically aggressive malignant disease suggested that *EGFr* was a promising target for several epithelial tumours, which represent approximately two thirds of all human cancers. Furthermore, the favourable interaction profile for *EGFr* blocking agents combined with radiation has stimulated clinical trials in diverse anatomical sites including head and neck, colorectal region, pancreas and lung [2], where molecular inhibition of *EGFr* signalling in combination with radiation represents a highly promising area [3,4].

Therefore, new radiobiology studies have focussed on identifying correlations between radiosensitization and biological agents. However, these effects have not been fully integrated into current radiobiological models [5-8]. One such model commonly used in clinical practice, is the *BED* obtained from the *LQ* model [9], given by the following equation (proliferation ignored):

(1) $$BED=D\cdot \left(1+\frac{d}{\alpha /\beta }\right)\text{,}$$

where *α* and *β* represent intrinsic and repair cell radiosensitivity, respectively, *d* represents the dose per fraction and *D* is the total dose delivered during the radiation treatment. The *BED* is considered a “biological dose” delivered by a particular combination of dose per fraction and total dose to a given tissue, characterized by a given *α/β* ratio, and is commonly used to equate or compare various fractionation schedules [10].

However, eq. (1) demonstrates that the same number of cells killed – the equivalent effect – could be obtained equating the *BED* not only for schedules with different numbers of fractions and various doses per fraction, but also for schedules where the dose per fraction is increased if a reduction in radiosensitivity results (i.e. *α* or *β* is reduced).

This could be applicable for subsets of cells that over-express *EGFr*, representing a source of heterogeneity closely connected with the repopulation rate and intrinsic radiosensitivity. However, the heterogeneous population of *EGFr* expression cannot be represented by a single equation of tumour control probability (*TCP*), as it is intrinsically linked to a group of tumours with identical characteristics [11].

Furthermore, equations considering the radiation response that take into account different compartments of sensitivity within tumours [12] or a Gaussian distribution of individual radio sensitivities [13,14] cannot be used because various levels of radiosensitivity coexist in the tumors or in the statistical sample.

Therefore, during this computational study, a new mathematical interpretation of radiosensitivity parameters that are normally used in standard radiobiological models (i.e. as functions of *EGFr* expression) is proposed using simple examples.

The final aim of the current study is to provide an additional mathematical tool that can be used to carry out radiobiological analysis, taking into account the radioresistance effects due to *EGFr* over-expression and/or radiosensitization effects due to *EGFr* inhibitors when they are combined with radiation.

These examples are not intended to simulate a particular type of radiotherapy treatment, but are designed to demonstrate a general effect.

## Materials and methods

**Figure 1:**
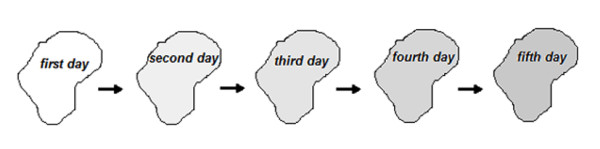
Schematic representation of radiosensitivity variability within a single tumour due to the presence of varying concentrations of radiosensitizer drugs (Light gray = high radiosensitivity, dark gray = low radiosensitivity).

During the current analysis two separate groups of patients with various levels of *EGFr* expression were considered. For each of the *EGFr* groups, various values for the parameters *α*, *β*, were considered. This approach allowed radiobiological analysis to be conducted in cases where differences in radiosensitivity occurred within the same tumour after combined treatments comprising radiation and radiosensitizing *EGFr* inhibitors [4,15,16]. In the latter case, various levels of radiosensitivity did not coexist, but they followed one another according to the concentration of radiosensitizing drug present during the radiotherapy session (Figure 1).

*Modified BED: Effects due to a change in EGFr expression levels EGFr* expression has been assessed through intensity of staining (i.e., absent, minimal, moderate, or intense staining) in clinical practice [17]. During the present analysis, normal and high expression levels of *EGFr* (i.e. below and above 50% staining) were distinguished. The subscript *H* was added to indicate high *EGFr* expression.

The *BED* for the *EGFr* group with high expression may be indicated as:

$$BED_{H}=n\cdot d\cdot \left(1+\frac{d}{\alpha_{H}/\beta_{H}}\right)$$

Here, because *α*_*H*_ and *β*_*H*_ are lower than *α* and *β* (reduced radiosensitivity), the number of cells killed with the same dose per fraction (*d*) and the number of fractions (*n*) were reduced with respect to standard radiosensitivity conditions. Therefore, the following inequality arose:

$$\alpha \cdot BED>\alpha_{H}\cdot BED_{H}$$

To obtain the same effect with an equal number of fractions, a change of dose/fraction is necessary. We introduce the *MBED*:

(2) $$MBED=n\cdot \delta \cdot \left(1+\frac{\delta }{\alpha_{H}/\beta_{H}}\right)$$

where the dose *δ*, which refers to *α*_*H*_ and *β*_*H*_, has the effect equivalent to *d*, which refers to *α* and *β*, so that:

(3) $$\alpha \cdot BED=\alpha_{H}\cdot MBED$$

In eq. (3) the LHS provides a measure of treatment effect under standard conditions of radiosensitivity, while the RHS represents the same effect achieved under non standard conditions of radiosensitivity.

From eq. (1), eq. (2) and eq. (3):

$$\alpha \cdot d\cdot \left(1+\frac{d}{\alpha /\beta }\right)=\alpha_{H}\cdot \delta \cdot \left(1+\frac{\delta }{\alpha_{H}/\beta_{H}}\right)\text{,}$$

and solving for *δ*

(4) $$\delta =-\frac{\alpha_{H}}{2\beta_{H}}+\sqrt{{\left(\frac{\alpha_{H}}{2\beta_{H}}\right)}^{2}+d\cdot \left(\frac{\alpha }{\beta_{H}}+\frac{\beta }{\beta_{H}}d\right)}$$

Therefore, the *MBED* distinguishes between changes in biological effect due to irreparable and/or reparable damage variations, rather than changes due to dose/fraction or total dose variations. A reduction in radiosensitivity due to increased membrane *EGFr* expression [11,18] implies equivalence between treatments by increasing the dose per fraction with an equal number of fractions.

Furthermore, to obtain isoeffective treatments with a different number of fractions *m* (*m* < *n* hypo-fractionation, *m* > *n* hyper-fractionation) from eq. (3), the following results:

(5) $$\delta =-\frac{\alpha_{H}}{2\beta_{H}}+\sqrt{{\left(\frac{\alpha_{H}}{2\beta_{H}}\right)}^{2}+\frac{n\cdot d}{m}\cdot \left(\frac{\alpha }{\beta_{H}}+\frac{\beta }{\beta_{H}}d\right)}$$

### Modified BED: Effects due to biological drugs

Combined treatment comprising radiation and radiosensitizing *EGFr* inhibitor drugs requires the daily dose that achieves the same effect without drugs to be calculated. This will result in a calculation of the daily radiosensitivity conditions induced by the drug compared with standard radiosensitivity.

On the basis of a preclinical assessment, we propose a method to estimate the daily radiosensitivity when radiotherapy treatment is combined with biological drugs. Subsequently, the *MBED* method is applied to assess the changes required in terms of dose fractionation when such daily radiosensitivity is considered.

**Figure 2:**
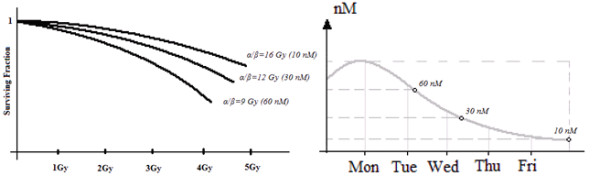
**First phase to investigate the effects *EGFr *over-expression on radiosensitivity of Head and Neck cell lines.** Data from literature [16,18,19] demonstrate the correlation between *EGFr* over-expression and reduced cellular radiosensitivity. This situation is indicated by an upward shift of the cell survival curve in the line over-expressing *EGFr* compared with normal *EGFr* expression. A polynomial regression allows radiosensitivity parameters corresponding to various surviving curves to be calculated.

During the first phase, survival curves obtained with various concentrations of a monoclonal antibody (mAb) *EGFr* inhibitor were selected from the literature [16,18]. From these curves, using a polynomial regression, the corresponding values of *α* and *β* were calculated (Figure 2(a)). However, the drug concentrations reported in these studies do not correspond to the effective drug concentrations used during the combined treatment with radiation every day of treatment (Figure 2(b)).

**Figure 3:**
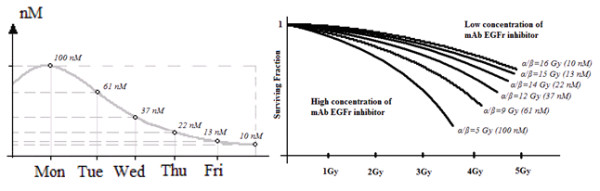
**Second phase to estimate the effects on radiosensitivity of variable concentrations of mAb *EGFr *inhibitor in Head and Neck cell lines.** Surviving fraction curves corresponding to the daily concentrations of mAb from pharmacokinetics curves [20] are obtained by interpolation. The following concentrations of *EGFr* mAb inhibitor are obtained: 100, 61, 37, 22 and 13 nM. The corresponding polynomial regression curves provide *α/β = 5, 9, 12, 14* and *15 Gy* (with *α = 0.2 Gy*^*-1*^), with respect to untreated cells with *α/β = 16 Gy* (*α = 0.2 Gy*^*-1*^).

Therefore, during the second phase, the daily in vivo concentration of *EGFr* inhibitor drug was calculated from its pharmacokinetic curve and drug dosage [20]. Referring to these daily concentrations, it is possible to interpolate plausible corresponding curves of survival fractions, obtaining the researched values of *α* and *β* using a new polynomial regression (Figure 3).

Subsequently, assuming a daily in vivo radiosensitivity, eq. (3) with a variable concentration of a radiosensitizing drug according to the weekly dosage can be written as follows:

$$n\cdot d\cdot \left(\alpha +\beta \cdot d\right)=n_{w}\cdot \left[\delta_{1}\cdot \left(\alpha_{1}+\beta_{1}\cdot \delta_{1}\right)+...+\delta_{5}\cdot \left(\alpha_{5}+\beta_{5}\cdot \delta_{5}\right)\right]$$

where *n*_*w*_ (*n*_*w*_ = *m/5*) represents the number of weeks of overall treatment and the numbers *1,2,…,5* indicate the day of the week. In compact form, we can write:

$$n\cdot d\cdot \left(\alpha +\beta \cdot d\right)=n_{w}\cdot \sum_{i=1}^{5}\delta_{i}\cdot \left(\alpha_{i}+\beta_{i}\cdot \delta_{i}\right)\text{,}$$

Therefore, an equivalent fractionation with the same number of fractions is obtained using the following:

(6) $$d\cdot \left(\alpha +\beta \cdot d\right)=\frac{1}{5}\sum_{i=1}^{5}\delta_{i}\cdot \left(\alpha_{i}+\beta_{i}\cdot \delta_{i}\right)\text{,}$$

From eq. (6), a solution with equal dose for each day is:

(7) $$\delta =-\frac{\sum_{i}\alpha_{i}}{2\sum_{i}\beta_{i}}+\sqrt{{\left(\frac{\sum_{i}\alpha_{i}}{2\sum_{i}\beta_{i}}\right)}^{2}+\frac{5\cdot d\cdot \left(\alpha +\beta \cdot d\right)}{\sum_{i}\beta_{i}}}$$

In addition, eq. (6) highlights the possibility of solutions with a dose adapted to the daily radiosensitivity. By equating the effect day to day during the week, for the *ith* day we obtain:

$$d\cdot \left(\alpha +\beta \cdot d\right)=\delta_{i}\cdot \left(\alpha_{i}+\beta_{i}\cdot \delta_{i}\right)\text{,}$$

therefore:

(8) $$\delta_{i}=-\frac{\alpha_{i}}{2\beta_{i}}+\sqrt{{\left(\frac{\alpha_{i}}{2\beta_{i}}\right)}^{2}+d\cdot \left(\frac{\alpha }{\beta_{i}}+\frac{\beta }{\beta_{i}}d\right)}$$

Eq. (8) leads to a modified fractionation modulated according to the pharmacokinetics of the drug combined with radiation. For a schedule with different numbers of fractions:

(9) $$\delta_{i}=-\frac{\alpha_{i}}{2\beta_{i}}+\sqrt{{\left(\frac{\alpha_{i}}{2\beta_{i}}\right)}^{2}+\frac{n\cdot d}{m}\cdot \left(\frac{\alpha }{\beta_{i}}+\frac{\beta }{\beta_{i}}d\right)}$$

This solution leads to a modulated hypo-fractionation if the number of weeks is less than the standard fractionation (vice versa for the hyper-fractionation).

Eq. (7) and eq. (9) represent dose values that have the same effect. However, as in the drug is also absorbed by normal tissue cells, these cells will show increased radiosensitivity. Therefore, modulated dose fractionation with a reduced dose of radiation corresponding to higher radiosensitivity could lead to a reduction in harmful effects.

This proposal could be verified through clinical trials.

## Results

This section discusses results from representative examples occurring in clinical practice. Schedules with the equivalent effect of *30* fractions of *2 Gy*/fraction (assumed as a reference standard regime) were calculated. To analyze an increase in radiosensitivity, a change in *α* or *β*, and consequently a change in *α/β,* has been assumed to simplify the calculations without losing generality.

For examples 3, 4 and 5, substantial changes in *β* alone has been adopted, assuming that data were obtained from the polynomial regressions of curves depicted in Figure 3.

Of note, the unchanged *α*, *β* (without polynomial regression) and the fractionation schemes assumed in these examples are plausible but should not be considered as recommendations for real clinical situations.

**Figure 4:**
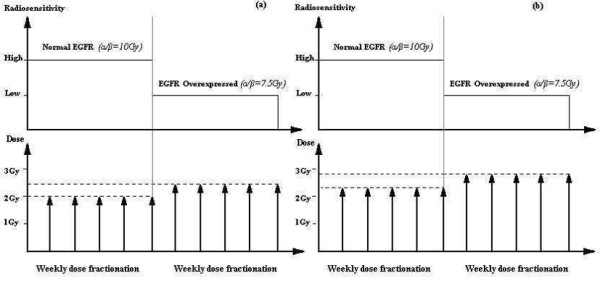
**Weekly dose/fraction as a function of radiosensitivity for modified fractionations with** (**a**) **same number of fractions as the reference fractionation (*Example 1*) and (b) hypo-fractionation with one week less than reference fractionation (*Example 2)*.**

**Figure 5:**
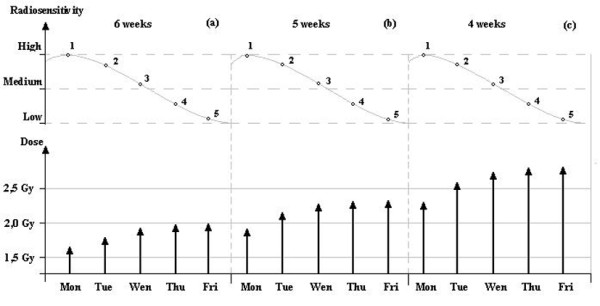
**Dose/fraction as a function of daily radiosensitivity for modulated fractionations with (a) same number of fractions as the reference schedule (*Example 3: 6 weeks*), (b) hypo-fractionation with one week less than the reference schedule (*Example 4: 5 weeks*) and hypo-fractionation with two weeks less than the reference schedule (*Example 5: 4 weeks*).** The grey lines represent radiosensitivity corresponding to the pharmacokinetics curves of absorption for the *EGFr* mAb inhibitor. Abbreviations: (1) *α/β* = 5 Gy; (2) *α/β* = 9 Gy; (3) *α/β* = 12 Gy; (4) *α/β* = 14 Gy; (5) *α/β* = 15 Gy.

Dose fractionations are presented for examples demonstrated in Figure 4 and Figure 5. These figures present the extent of dose for fraction as a function of weekly or daily radiosensitivity; tables 1 and 2 present numerical results.

**Table 1:** **Numerical results for *Examples 1 *and *2***

***EGFr *expression**	***α*(Gy**^**-1**^**)**	***β*(Gy**^**-2**^**)**	***α/β*(Gy)**	***d***_***ex1***_**(Gy)**	***d***_***ex2***_**(Gy)**
Normal	0.2	0.02	10	2.00	2.33
Over-expressed	0.15	0.02	7.5	2.42	2.80

Abbreviation: *d*_*ex1*_ and *d*_*ex2*_ = doses from *MBED* for *Example 1* and *2*, respectively.

**Table 2:** **Numerical results for *Examples 3, 4 *and *5***

**Day**	***α*(Gy**^**-1**^**)**	***β*(Gy**^**-2**^**)**	***α/β*(Gy)**	***d***_***ex3***_**(Gy)**	***d***_***ex4***_**(Gy)**	***d***_***ex5***_**(Gy)**
Monday	0.2	0.040	5	1.68	1.94	2.31
Tuesday	0.2	0.022	9	1.86	2.18	2.62
Wednesday	0.2	0.017	12	1.93	2.27	2.74
Thursday	0.2	0.014	14	1.98	2.32	2.81
Friday	0.2	0.013	15	1.99	2.34	2.84

Abbreviation: *d*_*ex3*_, *d*_*ex4*_ and *d*_*ex5*_ = doses from *MBED* for *Example 3, 4* and *5*, respectively.

### Example 1

In this example a selection of patients that should be treated with the reference schedule (consisting of *30* fractions of *2 Gy*/fraction on PTV) was assumed. Patients in the first subset (G1) were considered to have normal *EGFr* expression on clonogenic tumour cells, with radiosensitivity corresponding to *α = 0.2 Gy*^*-1*^, *β = 0.02 Gy*^*-2*^ (*α/β = 10 Gy*). In addition, we considered a second subset of patients (G2) as presenting with *EGFr* cell membrane over-expression, resulting in a reduction of radiosensitivity with *α*_*H*_ *= 0.15 Gy*^*-1*^, *β*_*H*_ *= 0.02 Gy*^*-2*^ (*α/β*_*H*_ *= 7.5 Gy*).

Therefore, with respect to the reference schedule, the effect for the subset G1 would be:

$$\alpha \cdot BED=0.2\cdot 30\cdot 2\cdot \left(1+\frac{2}{10}\right)=14.4$$

Owing to the reduction of the *α* component of irreparable damage, the same schedule used for group G2 will produce the following effect:

$$\alpha_{H}\cdot BED_{H}=0.15\cdot 30\cdot 2\cdot \left(1+\frac{2}{7.5}\right)=11.4$$

with a noticeable reduction in the effect of overall treatment.

To produce the same therapeutic effect for patients in G2 as received by patients in group G1 (with the same number of fractions taken in the reference treatment), the dose per fraction should be increased by imposing condition (3). Then, from eq. (4), we obtain:

δ=2.42Gy

To achieve the same effect on the PTV, *30* fractions of *2.42 Gy*/fraction should be given to compensate for reduced radiosensitivity due to over-expression of membrane *EGFr* (Table 1 and Figure 4).

The new schedule will be not equivalent in terms of toxicity to organs at risk (OAR). Therefore, the plan will require re evaluation of the harmful effects for OARs. In the opposing situation, that is for an increase of radiosensitivity in the clonogens of G2 compared with G1 (owing to a radiosensitizing drug), one can adopt the same procedure. In such cases, the equivalent effect on the PTV, with the same number of fractions, will be reached by reducing the fraction dose.

### Example 2

For the same subsets of patients used in *Example 1*, we analyzed a hypo-fractionated schedule that lasted for one week less for patients in G2, with the same effect as the standard schedule for patients in G1. In the hypo-fractionation schedule, the number of fractions was *m = 5·(n*_*w*_*-1) = 5·5 = 25* fractions.

Applying eq. (5) we obtain:

δ=2.80Gy

therefore, the hypo-fractionated schedule for patients in G2 will be equivalent to the standard schedule for patients in G1 if *25* fractions of *2.80 Gy*/fraction are given. If *α/β = 10 Gy* and a normal radiosensitivity is assumed, we would obtain:

$$0.2\cdot 30\cdot 2\cdot \left(1+\frac{2}{10}\right)=0.2\cdot 25\cdot d\cdot \left(1+\frac{d}{10}\right)\text{,}$$

from which:

d=2.33Gy

which would underestimate the dose required to achieve the same effect on the PTV (Table 1 and Figure 4).

### Example 3

In this example we refer to group G2 having substantial membrane *EGFr* over-expression, with *α*_*H*_ *= 0.2 Gy*^*-1*^ and *α*_*H*_*/β*_*H*_ *= 16 Gy* (similar estimated *α/β* values are reported in the literature [21]). We compare the reference treatment with a combined treatment comprising radiation and biological drugs that produce an increase in radiosensitivity.

In addition, we assume a weekly drug dosage with a pharmacokinetics curve showing maximum absorption during the first day of treatment [20]. The weekly radiosensitivity is assumed to be that described by the set of values reported in Table 2.

The equivalent treatment with the same number of fractions is obtained using eq. (7). In this case, a constant dose for each day is obtained, equaling the global effect.

δ=1.88Gy

Subsequently, using eq. (8), a dose modulated according to the drug pharmacokinetics is obtained, equaling the effect for each day of treatment. Results are presented in Table 2 and Figure 5.

### Examples 4 and 5

The equivalent global effect of the reference schedules could be obtained by subtracting one or two weeks of treatment from eq. (9), with a modulated soft hypo-fractionation (5 weeks) and with a modulated strong hypo-fractionation (4 weeks), respectively. The results are presented in Table 2 and Figure 5.

## Discussion

During practical applications of radiobiological models, the main difficulty is to decide which parameter values should be included in individual calculations. It is important to clarify that population based estimates of the *α/β* value represent averages, and that values are likely to vary between and within tumour types. It is clear that the assumption of a single value for *α* or *α/β* is a simplification and this could have a considerable impact on the predictive use of *BED* when deciding on dose fractionation [22].

However, recent knowledge concerning molecular mechanisms allows new developments to be explored and provides important information relating to the intrinsic radiosensitivity and fractionation sensitivity. Cell studies in vitro demonstrate that differences in radiosensitivity occur among cell lines derived from different types of tumours or from the same type of tumour, and during irradiation when combined treatments using radiation and radiosensitizing drugs are utilised [16,23-25].

These considerations may lead the way for new studies concerning evaluation of *α* and *β*, in which cellular radiosensitivity is modified using known concentrations of radiosensitizing drugs, as described in Figure 4 and Figure 5.

Therefore, the historical inability to distinguish among effects resulting in differences in radiosensitivity could be overcome through new knowledge concerning heterogeneity [26,27]. These effects are well known from preclinical studies, and could be used to reduce uncertainties and investigated through clinical trials [28]. The ideal situation could be to use assay methods to allocate patients to various treatment schedules on the basis of individual measurements of tumour cell radiosensitivity (for example, due to varied expression of *EGFr*) or absorption of drugs. This approach is expected to be applied in the foreseeable future.

On the basis of these considerations, a new method to interpret *BED* expression, named *MBED*, was introduced during this computational study to take account of intrinsic differences in radiosensitivity.

The requirement to introduce *MBED* arises because radiosensitivity is usually considered to be fixed for a cell type and constant during any radiation treatment. For this reason, *α* and *β* are considered fixed values with considerable uncertainty. Therefore, in the standard use of the *BED*, the hypothesis that one fractionation is equivalent to another underlies the assumption that the values of *α* and *β* are the same: to have the same effect – resulting in the same number of cells being killed – changing the dose per fraction, one must alter the number of fractions.

Herein, it is argued that for various values of radiosensitivity, the same number of cells can be killed with the same number of fractions by varying the dose per fraction. This requires identification of prognostic parameters such as the over-expression of *EGFr*, which allows the radiosensitivity of the individual patient to be classified and the most appropriate radiation dose fractionation to be identified.

The results of this study demonstrate that for a subset of patients presenting with *EGFr* cell membrane over-expression, resulting in reduced radiosensitivity with respect to a subset of patients with normal *EGFr* expression of clonogenic tumour cells, the dose per fraction should be increased to produce the same therapeutic effect with the same number of fractions taken in the reference treatment.

When radiation is combined with a biological drug that produces an increase in radiosensitivity, depending on the drug dosage, the equivalent treatment with the same number of fractions is obtained by a dose of radiation modulated according to drug pharmacokinetics.

The dose needs to be increased if the number of fractions is reduced.

In the examples reported herein, the absorption of *EGFr* inhibitors was considered for cancer cells alone. In general, cells of normal tissues also absorb the drug. In particular, *EGFr* is over-expressed in skin cells. Therefore, the effect of increased radiosensitivity will affect these cells, and modulated fractionations with a lower dose of radiation corresponding to higher radiosensitivity could lead to a reduction of harmful effects.

With *MBED*, this study was not intended to implement a finely tuned model based on accurate data obtained from preclinical analysis. The aim was to demonstrate the potential of the model and its malleability in terms of including further information that selective preclinical studies may provide [19].

In addition, previous analyses have depended on the validity of the LQ model, which has limitations. In particular, the LQ model used during this study does not include the time factor. In the generalized LQ model [5,10] the temporal factor is affected by differences in *EGFr* expression due to its influence on potential doubling time, *T*_*D*_[29-32]. This temporal factor can be particularly important when the *MBED* model is used to compare treatment schedules that differ in terms of overall treatment times, tumour control or acute effects (where time dependent repopulation may be important). The difference of doubling time between the High *EGFr* group and the Low *EGFr* group identified during the current study will be investigated further in new studies. This difference in terms of *T*_*D*_ can be transformed into an equivalent dose that would be required to offset the modified proliferation occurring in one day. The value of this equivalent dose can be taken into account during the previous analysis.

Overall, in practical applications of the *MBED* concept, there should be careful consideration of the relevant physical dose variations, the possible range of biological parameters and pertinent clinical factors. The prudent clinical oncologist should use *MBED* as a guide during clinical decisions rather than as an absolute indicator. The advice of acknowledged experts in radiobiological modelling should be sought in more complicated clinical situations.

Despite these limitations, the *MBED* model provides a valid means of accounting for modulated intrinsic radiosensitivity effects, which is preferable to neglecting them by using a biologically uncorrected physical dose.

Furthermore, the method is not intrinsically associated with the disease, and can be applied to any case by integrating traditional treatment plans and improving the overall radiotherapy performances in combined treatments comprising radiosensitizing drugs.

## Conclusion

During this computational study, the *MBED* method was introduced. The *MBED* provides a new tool to estimate the effects of heterogeneity on tumour radiosensitivity and to assess the dose per fraction required for increased tumour radiosensitivity due to *EGFr* over-expression. Where radiotherapy treatment is combined with radiosensitizing drugs, *MBED* suggests that the fraction sizes modulated according to drug pharmacokinetics will allow new schedules of dose fractionation to be more effective.

In conclusion, the *MBED* method could improve overall radiotherapy performances and be utilised to perform more appropriate radiobiological analysis, particularly when combined treatment comprising radiation and biological drugs is employed.

## Competing interests

The authors declare they have no competing interests.

## Authors’ contributions

PP developed the model and designed the study. BAJ, LS, BV, DA, MC, FB, GI checked the appropriateness of the study from oncology, radiotherapy and mathematical points of view. PP, RC, MC and LS compiled the manuscript and produced the graphical illustrations. AN, GS, MB and RO supervised the manuscript from radiobiological and clinical point of view. All co-authors approved the manuscript.
