# Correction: modelling the correlation between EGFr expression and tumour cell radiosensitivity, and combined treatments of radiation and monoclonal antibody EGFr inhibitors

**DOI:** 10.1186/1742-4682-9-37

**Published:** 2012-08-22

**Authors:** Piernicola Pedicini, Rocchina Caivano, Barbara A Jereczek-Fossa, Lidia Strigari, Barbara Vischioni, Daniela Alterio, Marta Cremonesi, Francesca Botta, Antonio Nappi, Giuseppina Improta, Giovanni Storto, Alba Fiorentino, Marcello Benassi, Roberto Orecchia, Vincenzo Fusco

**Affiliations:** 1 I.R.C.C.S. C.R.O.B Regional Cancer Hospital, Rionero in Vulture, Italy; 2 U.O. of Radiotherapy, I.E.O. European Institute of Oncology, Milan, Italy; 3 University of Milan, Milan, Italy; 4 Laboratory of Medical Physics and Expert Systems, Regina Elena National Cancer Institute, Rome, Italy; 5 U.O. of Radiobiology, C.N.A.O, Pavia, Italy; 6 Service of Medical Physics, I.E.O. European Institute of Oncolog, Milan, Italy; 7 Service of Medical Physics, Scientific Institute of Tumours of Romagna I.R.S.T, Meldola, Italy

## Correction

After publication of this work [1], we noted that we inadvertently failed to include the complete list of all coauthors. The full list of authors has now been added and the Authors' contributions and Competing interests section modified accordingly.

## Competing interests

The authors declare they have no competing interests.

## Authors’ contributions

PP developed the model and designed the study. BAJ, LS, BV, DA, MC, FB, GI and AF checked the appropriateness of the study from oncology, radiotherapy and mathematical points of view. PP, RC, MC and LS compiled the manuscript and produced the graphical illustrations. AN, GS, MB, RO and VF supervised the manuscript from radiobiological and clinical point of view. All co-authors approved the manuscript.
